# Nanostructured lipid carriers of ivermectin as a novel drug delivery system in hydatidosis

**DOI:** 10.1186/s13071-019-3719-x

**Published:** 2019-10-10

**Authors:** Ehsan Ahmadpour, Zahra Godrati-Azar, Adel Spotin, Roghayeh Norouzi, Hamed Hamishehkar, Sanam Nami, Peyman Heydarian, Saba Rajabi, Maryam Mohammadi, Gregorio Perez-Cordon

**Affiliations:** 1 Drug Applied Research Center, Tabriz University of Medical Sciences, Tabriz, Iran; 2 Infectious and Tropical Disease Research Center, Tabriz University of Medical Sciences, Tabriz, Iran; 3 Department of Pathobiology, Faculty of Veterinary Medicine, University of Tabriz, Tabriz, Iran; 4 Immunology Research Center, Tabriz University of Medical Sciences, Tabriz, Iran; 5 Department of Medical Parasitology and Mycology, School of Medicine, Qazvin University of Medical Sciences, Qazvin, Iran; 6 Food and Drug Safety Research Center, Tabriz University of Medical Sciences, Tabriz, Iran; 7 National Cryptosporidium Reference Unit, Public Health Wales Microbiology, Singleton Hospital, Swansea, UK; 8 Swansea University Medical School, Swansea University, Swansea, UK

**Keywords:** Hydatid cyst, Ivermectin, Scolicidal, Treatment, Nano lipid carriers, Apoptosis

## Abstract

**Background:**

The larval stage of the tapeworm *Echinococcus granulosus* is the causative agent of hydatid disease in humans. This zoonotic parasitic infection remains a major health problem in certain areas of the world where is still endemic. In view of the ineffectiveness of some drug treatments, the surgical removal of cysts remains the preferred treatment option together with the administration of albendazole and mebendazole. However, severe side effects of these drugs have been reported which demands developing new scolicidal agents that confer suitable efficacy and fewer side effects during surgery.

**Methods:**

To that purpose, in the present work we assessed the effectiveness of ivermectin (IVM), a macrocyclic lactone endectocide that has shown to be an effective nematocidal drug against other important parasitic infections. To overcome the limitations observed in some drug formulations and resistance, we used nano lipid carriers (NLCs) as a targeted and sustained drug delivery system for IVM. We evaluated the *in vitro* cestocidal and apoptotic effects of NLCs-loaded IVM *versus* IVM by quantifying the expression of caspase-3 mRNA.

**Results:**

We found that after 60 and 120 min of administration, 800 μg/ml and 400 μg/ml NLCs-loaded IVM induced 100% mortality, respectively. On the other hand, the 800 μg/ml of IVM induced 100% mortality rate 150 min after administration. Additionally, we found that NLCs-loaded IVM induced higher mRNA caspase-3 expression suggesting a more potent apoptotic effect on the parasite.

**Conclusions:**

These data suggest that NLCs-loaded IVM may be a promising alternative to current treatments although *in vivo* studies are needed.

## Background

Cystic hydatid disease (CHD), known as hydatidosis, is an important zoonosis both in humans and animals worldwide. The helminth *Echinococcus granulosus* is the causative agent whose definitive hosts are canids, such as dogs and foxes. The intermediate hosts (herbivorous or omnivorous mammals such as humans, domestic animals and wildlife species) can become infected with contaminated food, water or by direct contact with infected dogs [1, 2]. Africa, Europe, Asia, Central and South America are the endemic areas for CHD [3, 4]. Hydatid disease causes adverse and slowly developing cysts, which occur in the liver, lungs, spleen, brain, bone marrow, and rarely in other organs, and are often asymptomatic [1, 4, 5].

To date, the preferred treatment and definitive therapy for hydatid cysts is a surgical procedure. However, due to multiple lesions in various organs or the development of cysts in high risk organs, surgery is not always applicable. Furthermore, the results of surgical procedures have not always been successful and in some cases have been accompanied by anaphylactic shock, asthma and recurrence of the disease [6, 7]. Currently, chemotherapy and percutaneous aspiration, injection of chemicals and re-aspiration (PAIR) are alternative treatments for CHD [8]. Many efforts have been made to provide an effective drug for cystic hydatidosis. Up to now, different scolicidal compounds have been used in order to deactivate the contents of the cysts, but have been accompanied by adverse side effects, such as sclerosing cholangitis, necrosis of the liver and methaemoglobinaemia [6–8].

Two benzimidazoles, albendazole and mebendazole, are drug therapies routinely used for CHD before and after the surgery [9]; however, several side effects of these drugs have been reported such as thrombocytopenia, leucopenia, alopecia, embryotoxic and hepatotoxicity [10]. Hence, developing new scolicidal agents that confer suitable efficacy and fewer side effects is necessary to be used during surgery [6, 7]. Ivermectin (IVM), as a macrocyclic lactone endectocide, is an agonist of neurotransmitter and binds to gamma-aminobutyric acid (GABA) receptor. Abrogation of the nerve impulses culminates in paralysis and death of the parasite [11–13]. Nano lipid carriers (NLCs) are a delivery system composed of a solid matrix that contains liquid nano-fatty particles. These nanoparticles are approved by the Food and Drug Administration (FDA) and European Medicines Agency (EMA) and possess a controlled and continuous release capability, have a cellular dimension and are compatible with tissues and cells [14]. These nano lipid carriers produce nano-capsules containing the drug with higher permeability and concentrations and, there upon, resolves concerns such as side effects, low drug solubility in water and lack of adequate drug delivery to protoscoleces (PSCs) in cysts. In the present study, NLCs-loaded IVM were used against PSCs. The expression of caspase-3, which is one of the unknown pathways of immunity in the parasite, was studied in the PSCs.

## Methods

### Preparation of NLCs-loaded IVM

DL-lactide and glycolide were purchased from Sigma-Aldrich (St. Louis, MO, USA) and recrystallized with ethyl acetate. Stannous octoate (Sn(Oct) 2:stannous 2-ethylhexanoate), nano lipid carriers (molecular weight of 2000, 3000 and 4000), dimethyl sulfoxide, polyethylene glycol (PEGs) and poloxamer 407 were purchased from Sigma-Aldrich. Glyceryl palmitostearate (Precirol® ATO 5) was purchased from Gattefossé (Lyon, France). The NLCs-loaded IVM were prepared using the hot homogenization technique [15]. In this method, the IVMs was dissolved in ethanol and added to molten lipidic phase (precirol + myglyol) and mixed completely. Then, the aqueous phase containing the emulsifier was added dropwise to the lipidic phase at the same temperature under homogenization at 20,000× *rpm* for 20 min. The NLCs nanoparticles were then produced by solidifying the hot nanoemulsion by cooling down to room temperature.

### Characterization of nanoparticles

To assess the possible interaction between the drug and carrier, Fourier transform infrared (FTIR) spectroscopy was used. Lyophilized empty NLCs and drug-loaded NLCs were embedded in KBr pellets and FTIR spectra of samples were recorded using a FTIR spectrophotometer (Shimadzu, Kyoto, Japan). The scans were performed over a wave number range of 4000–400 cm^−1^. Particle size, polydispersity index (PDI) and zeta potential of synthesized nanoparticles were measured by dynamic light scattering (DLS) (Zetasizer-ZS, Malvern, UK). The surface morphology of the nanospheres during the incubation period was evaluated by scanning electron microscopy (SEM) (Phenom ProX, Phenom-World B.V., Eindhoven, Netherlands).

### PSC collection, culturing, and genotyping

Hydatid cysts of *E. granulosus* were collected from the livers of naturally infected sheep (East Azerbaijan, Northwest Iran). The hydatid fluid was aspirated and transferred into a test tube under sterilized conditions. The PSCs were centrifuged at 800× *rpm* for 5 min. The live PSCs were cultured in hydatid fluid treated containing 100 IU/ml penicillin and 100 μg/ml streptomycin, and were subsequently incubated at 37 °C [16, 17]. To determine the *E. granulosus* genotype, DNA extraction was performed with a commercial kit (DNG-plus™ solution; CinnaGen, Tehran, Iran) according to the manufacturer’s instructions. Polymerase chain reaction (PCR) was conducted to amplify the cytochrome *c* oxidase subunit 1 gene (*cox*1) in a volume of 20 μl, containing Taq DNA polymerase (1 U), deoxynucleotide triphosphate (dNTP, 250 μM), Tris-HCl (pH 9.0, 10 mM), KCl (30 mM), MgCl_2_ (1.5 mM), template DNA (50 ng) and 1 μl of each primer (10 pmol). Details of the primer sequences used for PCR were described previously [17, 18]. To identify the PSC genotype, the amplicons were directly sequenced (Pouya Gostar Gene, Tehran, Iran).

### Scolicidal assay

In the present study, various scolicidal concentrations of IVMs and NLCs-loaded IVM (50, 100, 200, 400 and 800 μg/ml) were tested at different exposure times (15, 30, 60, 120 and 150 min). To assess the viability of PSCs, 10 μl of 0.1% eosin dye was added to the 20 μl remaining pellet of PSC and mixed gently. Dead PSCs absorbed eosin, but live PSCs remained colorless. The percentages of dead PSCs were estimated by counting a minimum of 300 PSCs. In this study, phosphate buffered saline (PBS) and NLCs were used as negative controls [16].

### DNA fragmentation assay

DNA fragmentation occurring during apoptosis was analyzed by agarose gel electrophoresis. Briefly, 10^4^ PSCs treated with IVMs and NLCs-loaded IVM were exposed for 24 h. Then, the DNA extraction was performed by the DNGplus™ solution (CinnaGen, City, Iran). Finally, gel electrophoresis was performed using 4 μl of the DNA extract on a 1.5% agarose gel, stained and observed under UV light.

### Scanning electron microscopy

The treated PSCs were washed three times in PBS. Afterwards, PSCs were fixed in 3% glutaraldehyde for 12 h at room temperature. Following fixation, dehydration of the PSC was carried out in ascending concentrations of ethanol. Finally, processed samples were sputter-coated with gold and examined by SEM (Phenom ProX) [17].

### Isolation of total RNA and synthesis of complementary DNA (cDNA)

The total cellular RNA was isolated from the untreated and treated PSCs following 15 h of exposure time using an RNX Plus Kit (CinnaGen). The quality and quantity of isolated RNA were evaluated by a NanoDrop 2000c spectrophotometer (Thermo Fisher Scientific, Waltham, MA, USA). Subsequently, the RNA was reverse-transcribed into cDNA and used as the template for PCR amplification using a reverse transcriptase kit (Thermo Fisher Scientific).

### Primer designing and quantitative real-time polymerase chain reaction (qRT-PCR)

In order to evaluate the apoptotic effects of IVMs and NLCs-loaded IVM on the cultured PSC, the specific primers of *E. granulosus* caspase-3 gene were designed by the Oligo Analyzer v.3.1 tool based on reference accession numbers of AB306934 (EF-1α) and LK028577 (caspase-3). Primer sequences and cycling conditions have already been described [17]. Real-time PCR was performed to determine the level of caspase-3 messenger RNA (mRNA) expression. The real-time PCR was carried out in a 20 μl reaction volume, containing 1 μl of cDNA (50 ng), 1.5 mM of MgCl_2_, 125 μM of dNTP, 0.1 U/µl Taq DNA Polymerase, and 0.5 pmol of specific primers.

### Statistical analysis

GraphPad PRISM v.5 (GraphPad Software, La Jolla, CA, USA; http://www.graphpad.com) was used to draw graphs and conduct the statistical analysis. The normality of scale variables was evaluated by the Kolmogorov–Smirnov test. Differences between the test and control groups for nominal variables were analyzed using the Mann–Whitney U-test and one-way analysis of variance (ANOVA). A *P-*value < 0.05 was considered as statistically significant and data were represented as the mean ± standard error of the mean.

## Results

### Genotyping of *E. granulosus* PSCs

To determine the genotype of the *E. granulosus* PSCs, PCR amplification by targeting the cytochrome *c* oxidase subunit 1 gene (*cox*1) was performed. Based on sequencing analysis, the G1 genotype (sheep strain) was confirmed (Additional file 1: Figure S1).

### SEM micrograph and zeta potential

**Fig. 1:**
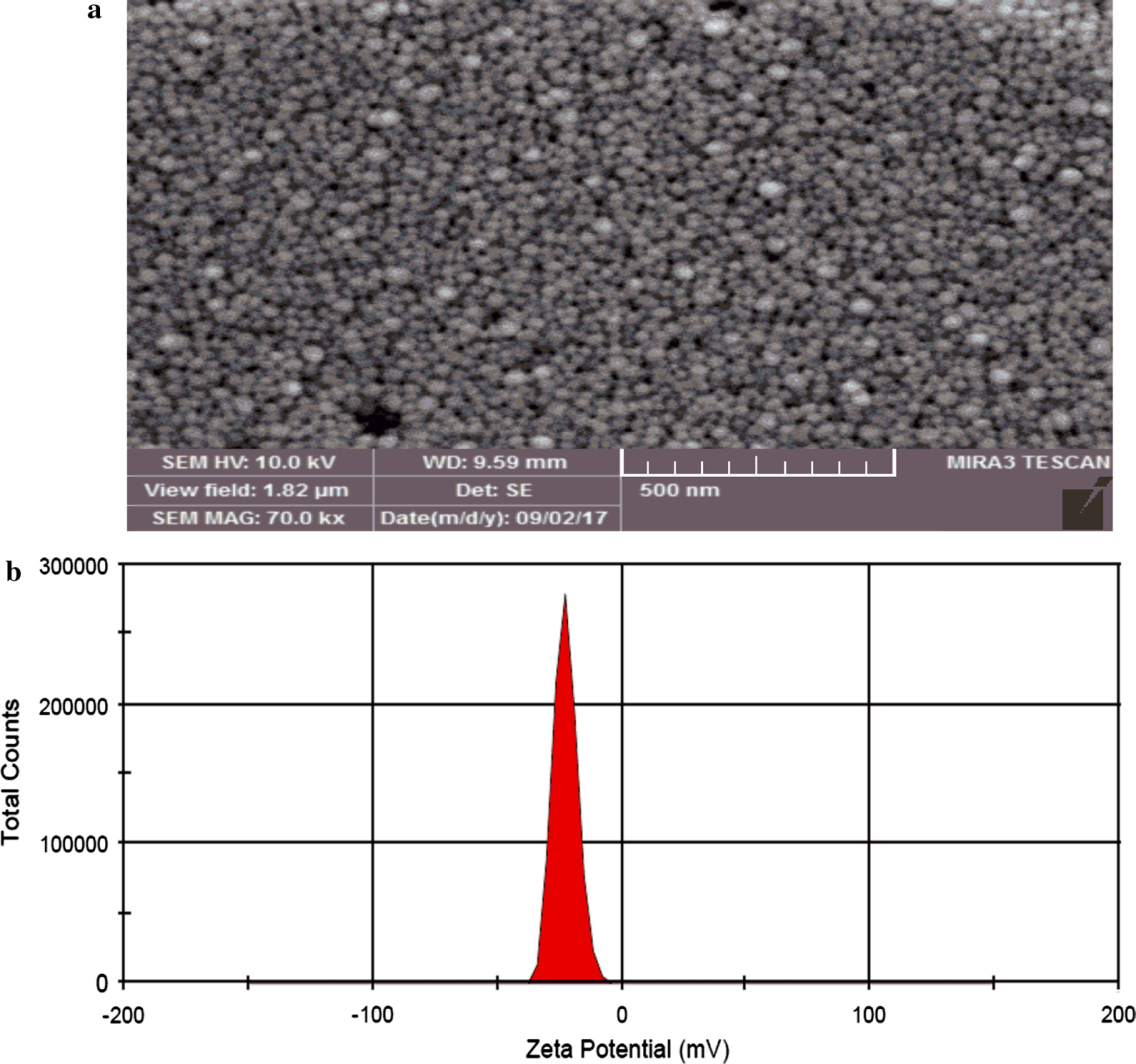
**a** SEM micrograph of NLCs-loaded IVM. **b** Zeta potential determination of NLCs-loaded IVM nanoparticles

The SEM micrograph of IVM-loaded NLC shown in Fig. 1a indicates that the polymeric nanoparticles were well distributed in the solution. In addition, the zeta potential distribution is also shown in Fig. 1b.

### Scolicidal effects of IVMs and NLCs-loaded IVM

**Fig. 2:**
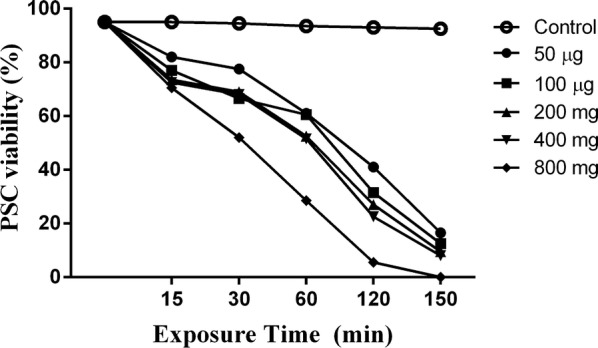
Scolicidal effects of IVMs against PSCs of *E. granulosus* at different concentrations following various exposure times (ANOVA: *F*_(1.2, 6)_ = 10.24, *P* = 0.0164)

**Fig. 3:**
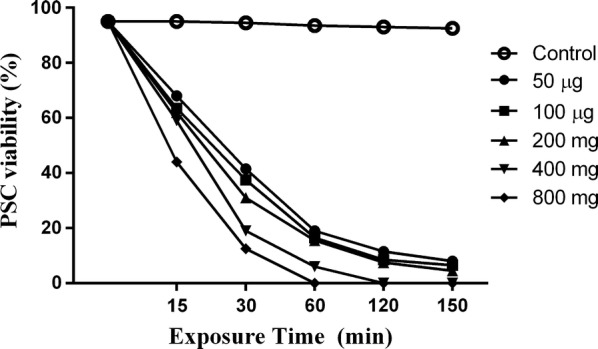
Scolicidal effects of NLCs-loaded IVM against PSCs of *E. granulosus* at the different concentrations following various exposure times (ANOVA: *F*_(1.1, 5.6)_ = 16.69, *P* = 0.0065)

**Fig. 4:**
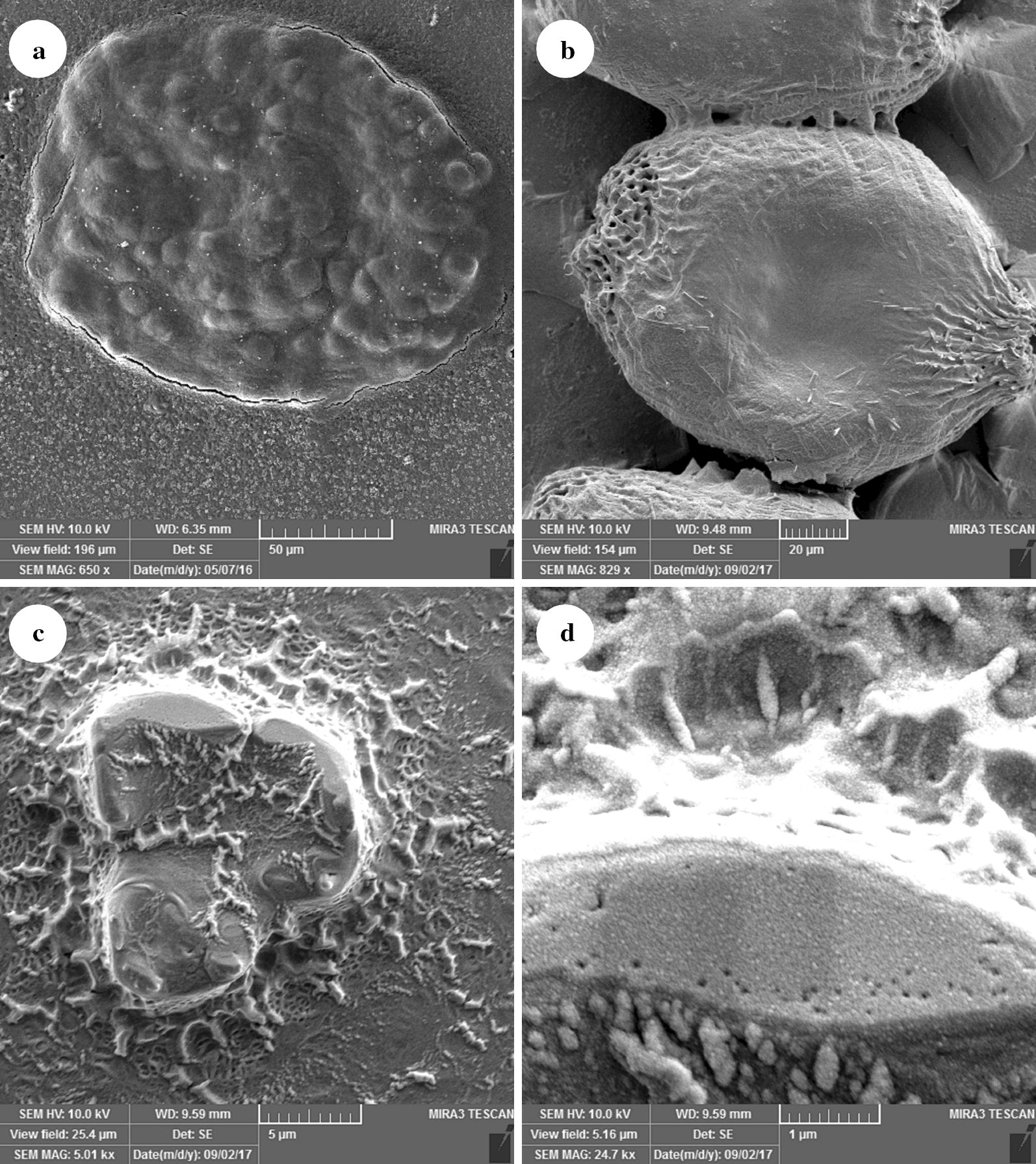
Ultrastructural morphology of treated PSC after 30 min using scanning electron microscopy (SEM). Protoscolices of *E. granulosus* treated with PBS (**a**), 50 μg/ml IVMs (**b**), 50 μg/ml NLCs-loaded IVM (**c**), and a zoomed image of a damaged protoscolex (**d**) (**a**, **b** invaginated protoscolces; **c** evaginated protoscolex)

The scolicidal effects of various concentrations of IVMs and NLCs-loaded IVM (50, 100, 200, 400 and 800 μg/ml) were tested at different exposure times (15, 30, 60, 120 and 150 min) (Additional file 2: Figure S2). Statistically significant differences were observed between 800 μg/ml IVMs at exposure times of 120 and 150 min (mortality rates of 96% and 100%, respectively) and the other concentrations and control group (PBS) (ANOVA: *F*_(1.2, 6)_ = 10.24, *P* = 0.0164; Fig. 2). However, the 50 μg/ml IVMs had an increasing scolicidal effect between 15 min (mortality rate of 20%) and 150 min (mortality rate 80%; Fig. 2). Remarkably, 800, 400 and 200 μg/ml of NLCs-loaded IVM could reach 100% mortality in 60, 120 and 150 min, respectively. These results were statistically significant compared with the other concentrations and control group (ANOVA: *F*_(1.1, 5.6)_ = 16.69, *P* = 0.0065; Fig. 3). The 50 μg/ml IVMs-loaded NLC was able to yield an increasing scolicidal effect between 15 min (mortality rate of 35%) and 150 min (mortality rate of 96%) of exposure (Fig. 3). The ultrastructural morphology of PSCs treated by NLCs-loaded IVM is shown in Fig. 4.

### Evaluation of caspase-3 mRNA expression and DNA fragmentation

**Fig. 5:**
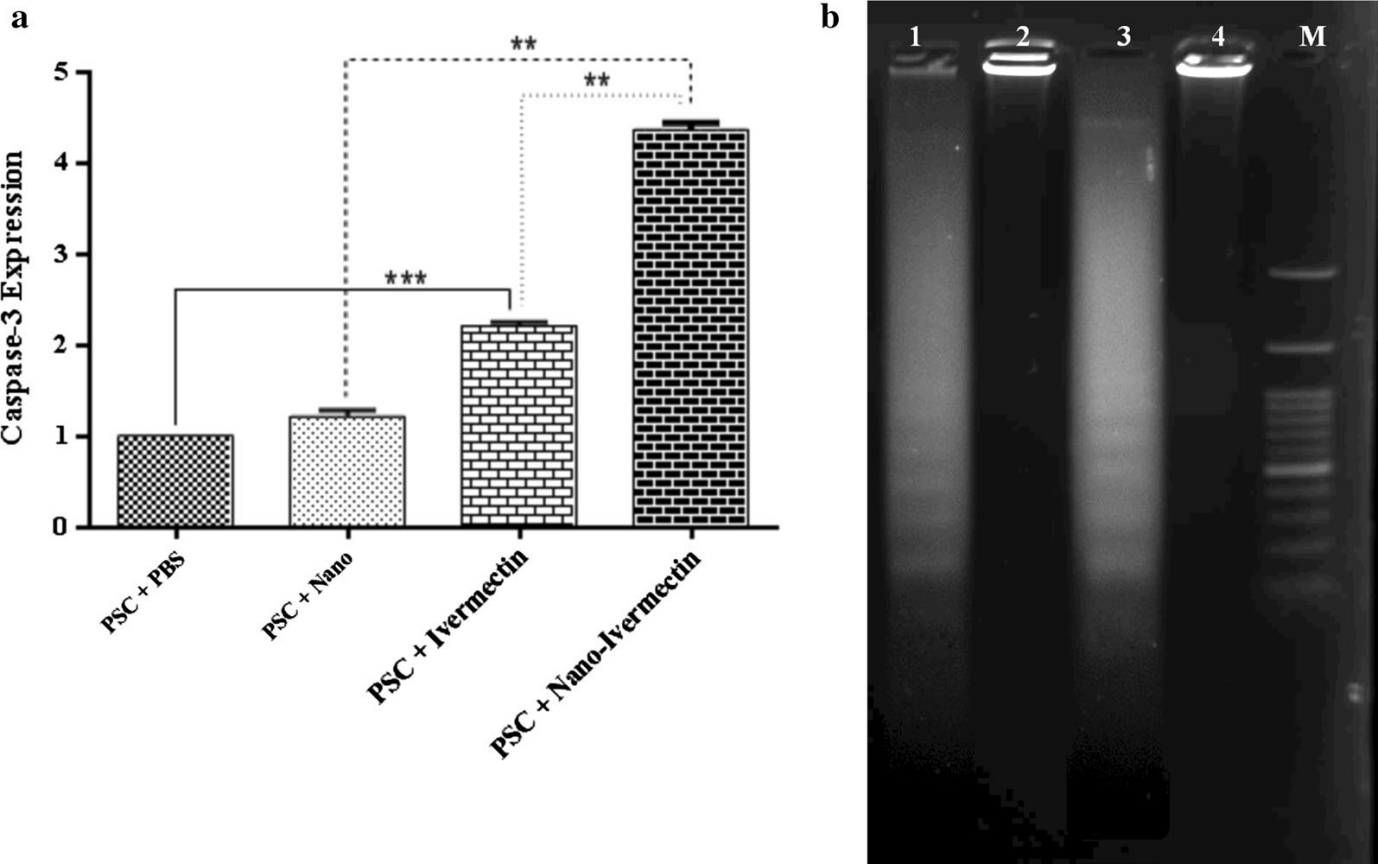
**a** An expression of caspase-3 mRNA level in PSCs treated with PBS (negative control), PSCs treated with NLCs (negative control), PSCs treated with IVMs, and PSCs treated with NLCs-loaded IVM. Bar graph indicates the mean ± standard deviation. Caspase-3 mRNA expression was higher in both PSC treated with IVMs and NLCs-loaded IVM than the control groups (PBS, Mann–Whitney U-test: *Z* = − 2.80224, *P* = 0.005; NLCs, Mann–Whitney U-test: *Z*  = − 2.50672, *P* = 0.01). **b** Protoscolex DNA fragmentation after treatment with IVMs (Lane 1), PBS (Lane 2), NLCs-loaded IVM (Lane 3), NLCs (Lane 4) and a 100-bp DNA marker (Lane M)

In this study, apoptosis was assessed by caspase-3 mRNA expression and DNA fragmentation assay. The expression of caspase-3 mRNA was assessed by the qRT-PCR after 15 h exposure (Fig. 5). Caspase-3 mRNA expression was higher in both PSC treated with IVMs and PSC treated with NLCs-loaded IVM relative to the control groups (PBS, Mann–Whitney U-test: *Z*  = − 2.80224, *P* = 0.005; and NLCs, Mann–Whitney U-test: *Z* = − 2.50672, *P* = 0.01); however, the apoptosis rate was significantly different (Mann–Whitney U-test: *Z* = − 2.64, *P* = 0.015) between the PSCs treated with IVMs and IVM-loaded NLCs.

## Discussion

In the present study, aiming to find an alternative efficient treatment, we evaluated the *in vitro* cestocidal and apoptotic effects of NLCs-loaded IVM based on the nematocidal effectiveness of this compound. Treatment and management options for CHD include surgery, percutaneous treatment, antiparasitic drug treatment and monitoring [19]. Among them, surgery is the preferred approach; however, some unpredicted outcomes of surgery, such as anaphylactic shock, recurrence of disease and locating cysts in risky tissues, have made the application of alternative options necessary (such as chemotherapy) [20]. To date, benzimidazoles derivatives, such as mebendazole and albendazole, are recommended chemotherapy drugs [9]. However, due to side effects and the poor water-solubility of benzimidazoles, a new therapy is needed; the ideal therapy for CHD should provide maximum treatment efficiency and minimize the adverse side effects [21, 22]. In addition, other antiparasitic drugs, such as praziquantel and nitazoxanide, occasionally have also been used against hydatic cysts in animals and humans [23, 24]. IVM, commercially available as oral suspensions for small ruminants, dogs and humans, has been reported to be effective against human strongyloidiasis, filariasis, cutaneous *larva migrans* and intestinal nematodes including *Ascaris lumbricoides*, *Trichuris trichiura* and *Enterobius vermicularis* [13, 25]. The activity route of IVM is related to glutamate-gated chloride channels. Upon binding, IVM paralyzes the body-wall muscles and more-potent effects occur on the pharyngeal musculature [26]. Besides nematocidal activity, *in vitro* cestocidal effects of IVM have also been reported [27]. Campbell et al. [28] reported that IVM had no effects on cestodes whereas Casado et al. [29] found a significant *in vitro* effect on the hydatid cyst PSCs. Although using a different approach, our results confirmed *in vitro* protoscolicidal effect of IVM. The differences in evaluation approaches in other studies might be the cause of conflicting results. In this regard, after *in vitro* incubation of *E. granulosus* PSCs with IVM, Martínez et al. [30] suggested some changes in expression level of heat-shock proteins (HSP) (decreased hsp70 and increased hsp60 levels). Furthermore, Pérez-Serrano et al. [31] proposed a tegumental depolarization of the PSCs incubated *in vitro* with IVM that can be essential for parasite survival. However, some limitations observed in the strategy of formulations and drug resistances have encouraged the researchers to improve formulation lies in the state-of-the-art delivery systems. Recently, some studies have shown that nanomedicines offer new insight into the development of existing management strategies and can enhance both the solubility and stability of drugs [14, 32]. NLCs are a new generation of lipid nanoparticles that have been developed through the combination of advantages from different nanocarriers. This kind of carrier seems to be a suitable delivery system intended for topical, oral, pulmonary, ocular and parenteral administration of drugs [33–35]. We based our study on the successful effect of IVM against PSCs from previous studies. For example, Casado et al. [36] showed a more rapid scolicidal effect of IVM, so that no cyst development was observed in mice inoculated with PSCs (100 µg for 72 h). Furthermore, the study showed that after 16 h of exposure to 100 µg of IVM, a rostellar disorganization occurred and a complete paralysis of PSCs was observed after 48 h. Ochiengʼ-Mitula et al. [37] investigated IVM during per-cutaneous drainage of *E. granulosus* hydatid cysts. Results of this study showed that injection of IVM into a cystic hydatid caused cyst collapse and death of PSCs with ultrastructural changes on the germinal layer. Thus, based on the probed advantages of NLCs and the effectiveness of IVM, NLCs-loaded IVM were synthetized to overcome cyst wall barriers. The results of the present study were significant: NLCs-loaded IVM showed more efficient activity in comparison to IVMs, causing up to 100% mortality. Similar studies have used nanoparticle derivations as a novel and alternative treatment approach. Moazeni et al. [38] studied the *in vitro* and *in vitro* antihydatid activity of a nano emulsion of *Zataria multiflora*, a native flowering plant from southwestern Asia, observing an 88–100% scolocidal effect after 20 minutes of exposure. Rahimi et al. [16] showed a high scolicidal effect of biogenic Ag-NPs, in which the 0.15 mg/ml concentration caused a 90% mortality rate after 120 minutes of exposure [16]. In the other similar *in vitro* study of fungal chitosan isolated from *Penicillium* spp., 100% mortality was observed after incubating with 400 µg/ml for 180 minutes [39]. Fungal chitosan seems to have stronger scolicidal effects due to beneficial characteristics, such as easy access, low-cost culture, nutrient and processing; however, further *in vivo* and experimental studies are still needed to attain precise results [40]. *In vitro* and *ex vivo* activity of *Melaleuca alternifolia* oil (tea tree oil, TTO) at 20 mg/ml indicated 90% protoscolicidal action in *E. ortleppi* hydatid cysts at five minutes [41]. Mahmoudvand et al. [42] showed that *Zataria multiflora* Boiss essential oil caused a significant scolicidal effect (100%) at the concentrations of 12.5 and 6.25 μl/ml after 5 and 20 minutes of exposure, respectively [42].

There are two types of hydatid cysts in the intermediate hosts, namely fertile and infertile cysts. One of the ways to cause infertilization of the cysts is through induction of an apoptotic cascade by the host. Fertile hydatid cysts, due to the presence of PSCs and antigens 5, B, cyclophilin and Δ/β1 prolongation factor, play an important role in the development of anaphylactic reactions of hydatid cyst during surgery [43, 44]. Therefore, it seems necessary to design approaches to induce cyst infertility *in vivo*.

The innate immunity of humans against the hydatid cyst fluid and the hydatid cyst layers, especially the germinal layer, is one of the possible mechanisms for suppressing hydatid cysts as well as the infertility of fertilized cysts [45–47]. The new pathways of innate immunity of host against hydatid cysts include inflammasome, toll-like receptor (TLR) and apoptosis. Apoptosis has recently been established to be a major component of the host’s innate immunity in suppressing parasites [45, 48]. Apoptosis is programmed cell death carried out by a series of biochemical and morphological events, such as chromatin compression, DNA fragmentation and expression of intracellular and extracellular apoptotic molecules [48]. It is characterized primarily by DNA fragmentation, formation of apoptotic bodies and cysteine aspartate-specific protease activation. Apoptosis mainly occurs through two pathways, an extrinsic pathway belonging to the death receptor and the intrinsic pathway through mitochondria. These two apoptotic pathways come together in a same terminal termed execution pathway, which is initiated by activation of caspase-3 and finished by cell death [45, 48]. The central components of this machinery are caspases proteins. Caspase-1 and caspase-3 are the two cysteine-aspartic acid proteases that play central roles in the execution-phase of apoptosis [48, 49]. Studies have reported a relation between the drug-induced apoptosis rate and high level of caspase-1 and caspase-3 expression in PSCs from *E. granulosus* [17, 49].

## Conclusions

In the present study, a significant difference was observed in the scolicidal effects and apoptosis rate between PSCs treated with IVMs and PSCs treated with IVM-loaded NLCs. *In vivo* studies of NLCs-loaded IVM and further similar studies are still required to develop more effective IVM nano-compounds and provide a deep understanding of reactions between NLCs-loaded IVM and PSCs.

## Supplementary information

**Additional file 1: Figure S1.** Confirmation of the PSCs G1 genotype (*) *via* the *cox*1 gene in this study. **a** PCR (Lane 1: marker 100 bp; Lane 2: negative control; Lane 3: PSCs extracted DNA). **b** Maximum-likelihood phylogenetic tree for *E. granulosus* isolates, based on the *cox*1 gene using *Taenia multiceps* as the outgroup.

**Additional file 2: Figure S2.** Light microscopy micrographs of *E. granulosus* protoscolices after staining with 0.1% eosin. **a** Live protoscolex (colorless). **b** Dead protoscolex (red). **c** Lysed protoscolex.

## Data Availability

Data supporting the conclusions of this article are included within the article and its additional files.

## Abbreviations

CHD: cystic hydatid disease
IVM: ivermectin
NLCs: nano lipid carriers
PAIR: percutaneous aspiration, injection of chemicals and re-aspiration
SEM: scanning electron microscopy
